# The Potential for Gut Organoid Derived Interstitial Cells of Cajal in Replacement Therapy

**DOI:** 10.3390/ijms18102059

**Published:** 2017-09-26

**Authors:** Jerry Zhou, Michael D. O’Connor, Vincent Ho

**Affiliations:** 1 School of Medicine, Western Sydney University, Campbelltown, NSW 2560, Australia; M.OConnor@westernsydney.edu.au (M.D.O.); v.ho@westernsydney.edu.au (V.H.); 2 Medical Sciences Research Group, Western Sydney University, Campbelltown, NSW 2560, Australia

**Keywords:** stem cell, organoid, interstitial cells of Cajal, motility disorder

## Abstract

Effective digestion requires propagation of food along the entire length of the gastrointestinal tract. This process involves coordinated waves of peristalsis produced by enteric neural cell types, including different categories of interstitial cells of Cajal (ICC). Impaired food transport along the gastrointestinal tract, either too fast or too slow, causes a range of gut motility disorders that affect millions of people worldwide. Notably, loss of ICC has been shown to affect gut motility. Patients that suffer from gut motility disorders regularly experience diarrhoea and/or constipation, insomnia, anxiety, attention lapses, irritability, dizziness, and headaches that greatly affect both physical and mental health. Limited treatment options are available for these patients, due to the scarcity of human gut tissue for research and transplantation. Recent advances in stem cell technology suggest that large amounts of rudimentary, yet functional, human gut tissue can be generated in vitro for research applications. Intriguingly, these stem cell-derived gut organoids appear to contain functional ICC, although their frequency and functional properties are yet to be fully characterised. By reviewing methods of gut organoid generation, together with what is known of the molecular and functional characteristics of ICC, this article highlights short- and long-term goals that need to be overcome in order to develop ICC-based therapies for gut motility disorders.

## 1. Introduction

Developments in regenerative medicine, particularly in the last decade, have focused on generating and characterising intestinal organoid units; i.e., three-dimensional (3D) clusters of epithelial and mesenchymal cells generated from stem cells [1]. Organoid units can now be grown to contain all gut cell layers and demonstrate mature intestinal capacities, like digestion, peptide absorption, and peristalsis [2,3]. Thus, these gut organoid systems might potentially provide a long-term source of gastrointestinal cell types, such as interstitial cells of Cajal (ICC) and enteric neurons for disease modelling, drug discovery, and clinical transplantation therapy. Such a source of normal or diseased human ICC (either in isolation or as part of 3D gut organoids) could provide an unprecedented opportunity to investigate how different types of intestinal neural elements behave when they are reintroduced to the body, after their purification, expansion or manipulation ex vivo. This review investigates the possibility of using intestinal organoid-derived ICC to elucidate molecular mechanisms of motility disorders, and potentially, to repair injured intestine in human diseases. We present an overview of organoid culture systems and isolation methodologies of ICC. We also discuss challenges and future directions of ICC research in regenerative therapy.

## 2. ICC Description

The gastrointestinal (GI) tract generates two types of motility; the first type is characterised by stimulation-activated contractions that propagate a bolus along the GI tract, called peristalsis, while the second type consists of spontaneous cycles of slow wave contractions that do not involve bolus movement. ICC have been established as an essential cell type in the regulation of both types of GI motility [4,5,6]. Several types of ICC have been described based on their anatomical location and function (Table 1). ICC located around the circumference of the myenteric plexus are called ICC of the myenteric plexus (ICC-MY). These multipolar cells, with branched processes connecting each other, are thought to generate and propagate electrical slow waves [4,7,8]. The ICC and smooth muscle cells in the stomach are electrically coupled in situ, allowing for slow waves originating from ICC to conduct to smooth muscle cells [9]. This coupling between ICC and smooth muscle cells can be mediated by gap junctions [10] or by close apposition contacts [11]. ICC found in the circular muscle and longitudinal muscles (ICC-IM) are bipolar spindle-shaped cells aligned directionally with the surrounding smooth muscle cells. ICC-IM play a critical role in motor neurotransmission between the enteric nervous system and smooth muscle cells [12,13]. A third class of ICC, the deep muscular plexus ICC (termed ICC-DMP) are only found in the small intestine and are thought to be a specialised version of ICC-IM. ICC-IM and ICC-DMP are densely innervated by excitatory motor neurons [14] and inhibitory motor neurons [15,16]. Disruption or loss of ICC has been implicated in a host of GI motility disorders, including achalasia [17,18], slow transit constipation [19,20], intestinal pseudo-obstruction [21,22], Crohn’s disease [23], inflammation [24,25], and diabetic gastroparesis [26,27,28,29], as well as altered gut motility associated with aging.

**Figure 1:**
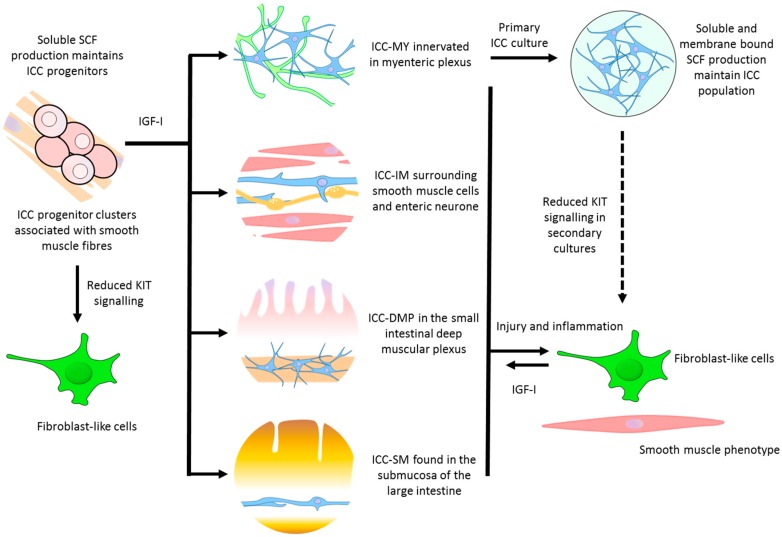
Possible pathways for maintenance, repair, and regeneration of interstitial cells of Cajal (ICC), IGF-I, insulin-like growth factor I; SCF, stem-cell factor. Solid arrows indicate defined differentiation pathways, dashed-arrow indicate potential differentiation pathway.

The mechanisms underlying ICC maintenance and turnover in vivo remain controversial, however, it is well recognized that ICC display a high degree of plasticity and regenerative capacity (Figure 1). ICC restoration has been observed in a variety of models, including immuno-neutralization [37,38], partial mechanical obstruction [39], surgical lesion [39,40], and inflammation [41]. These investigations showed that disruption of ICC networks can cause them to transdifferentiate into fibroblast/smooth muscle phenotype. Upon removal of the insult, these “transdifferentiated ICC” can then transdifferentiate back into ICC, which is associated with restoration of contractility.

During normal development of the small intestine, ICC have been shown to incorporate bromodeoxyuridine (BrdU), a synthetic thymidine analogue, suggesting that ICC can undergo cellular division to increase ICC numbers [42]. The incorporation of BrdU in ICC decreased with age, but was still observed in mice as old as 24 days, suggesting that division of ICC can occur in somewhat mature animals [43]. Thus, potential strategies to increase ICC numbers might be achieved through an increase in the survival of resident ICC, by stimulating ICC development from precursor cells, and by increasing proliferation. A recently discovered ICC population expressing CD34, but low levels of Kit in the *Tunica muscularis* of the gut, may represent progenitor ICC, that when properly stimulated, are capable of regeneration [30]. ICC can also be induced to proliferate by several molecules, including steel factor activation of the Kit receptor, neuronally derived nitric oxide, serotonin through the serotonin receptor 2B (5-HT2B receptor), and heme oxygenase-1 [44,45]. The plasticity and ability to self-renew are characteristics that make ICC an attractive candidate for regeneration and/or replacement therapy in patients.

## 3. Generation of Gut Organoids and ICC

Early sources of ICC were isolated from gut muscle strips or explant tissue cultures [46,47]. This approach involved processing strips of GI muscle via enzymatic dissociation, and subsequently, passing the cell suspension through progressively smaller (500–100 µm) filters to obtain a single cell suspension [48]. The resulting mixed cell population is seeded into culture plates and grown in smooth muscle growth medium. Whilst these explant cultures possess some “organotypic properties”, such as 3D architecture and cellular heterogeneity, they do not reproduce critical functional interactions between cell types of different germ layers; they are also limited to short-term culture.

The advent of stem cell derived organoids has offered the opportunity to produce a more complex 3D representation of a “mini gut” model for long-term research and potential clinical applications.

One of the first reports of stem cell-derived gut organoids was published in 2002 using mouse embryonic stem cells [49,50]. Using a combined non-adherent (“embryoid body”) and adherent culture, Kit^+^ ICC and protein gene product 9.5 (Pgp9.5^+^) enteric neurons networks were confirmed by immunohistochemistry within 14–21 days, which also correlated with the initial onset of electrical rhythmicity. A few years later, similar gut organoids were generated from mouse induced pluripotent stem cells (iPSC) [51], a pluripotent cell type established by forced expression of specific transcription factors in somatic cells. This process, termed cell reprogramming [52,53,54], offers the opportunity to make disease-specific human iPSCs (and therefore human gut tissue) from patients, to model the mechanisms of gut disorders and to perform drug discovery. In future, reprogramming may also provide an avenue for making patient-specific or human leukocyte antigen (HLA)-matched gut tissue for clinical applications.

Towards these ends, human iPSC cells have more recently been used to produce organoid intestinal tissue [55,56]. Spence et al. demonstrated that human iPSCs can be efficiently directed to differentiate in vitro into cell aggregates with 3D architecture and cellular composition, similar to human fetal intestinal tissue. Although these organoids were complex and contained multiple cell lineages, they lacked many of the cellular inputs present in an in vivo system (e.g., neural, endothelial, or immune cells). Watson et al. took this concept further, by establishing an in vivo human intestinal organoid model by engrafting 6-week old human iPSC organoids onto mouse kidney to generate mature, functional human intestinal tissue that responds to physiological stimuli. The human intestinal organoids underwent considerable maturation following in vivo engraftment compared to the previous ex vivo organoids models. Functionally, engrafted organoids expressed active brush border enzymes and were capable of peptide uptake [56].

One of the main challenges of generating functional organoids has been development of innervation by cells representing the enteric nervous system (ENS). A report from Workman et al. in 2016 used principles of embryonic intestinal development to combine human iPSC derived enteric neural progenitors with iPSC derived intestinal organoids to form in vitro functional human intestinal tissue. Neural progenitors introduced into organoids migrated into the mesenchyme, self-organised, and differentiated into neurons and glial cells of the ENS. The functionality of the ENS within the organoid was confirmed by the detection of rhythmic waves of calcium transients. After engraftment and in vivo growth of these ENS-organoids, they formed neuroglial structures, similar to myenteric and submuscosal plexus, they had functional ICC, and also an electromechanical coupling that regulated waves of propagating contractions [2].

To date, human gut organoid systems have used sequential differentiation induction steps using a panel of growth factors. These steps are designed to first generate endoderm while suppressing formation of ectodermal and mesodermal cell types, and then induction of differentiation into specific intestinal cell types [55]. In doing so, it can be argued that these protocols do not mimic the functional interactions between cells of the three germ layers that occur during organogenesis in vivo. Moreover, in these studies, differentiation occurred under xenogeneic condition, such as the use of fetal bovine serum, which complicates the possible use of these structures in clinical applications. In an attempt to address these issues, in 2017, Uchida et al. published a study in which they used human iPSC cells and xeno-free conditions to generate the first functional intestinal organoid containing three germ layers [3]. These gut organoids were composed of intestinal epithelium and mesenchymal layers containing KIT^+^ ICC. Immunostaining identified ICC-like cells within the myenteric and submucosal plexuses. Peristalsis-like movement was observed from day 80, and the organoids responded to the contractile modulators, histamine and atropine. Importantly, unlike the other organoids grown in vitro, these gut organoids display functional characteristics of mature intestinal tissue. In addition to peristalsis, the presence of both intestinal epithelium and mesenchymal layers allowed for peptide absorption and fluid secretion. Despite these promising results, motility was only observed in a small portion of the organoids (4%), with immunostaining suggesting immaturity of some cells within the mesenchymal layer, such as smooth muscles and neurons. Further improvements will be needed to generate in vitro gut organoids with mature mesenchymal layers and full motility and the properties of key motility-related types (such as ICC) need to be more fully characterised.

## 4. Current ICC Makers

**Figure 2:**
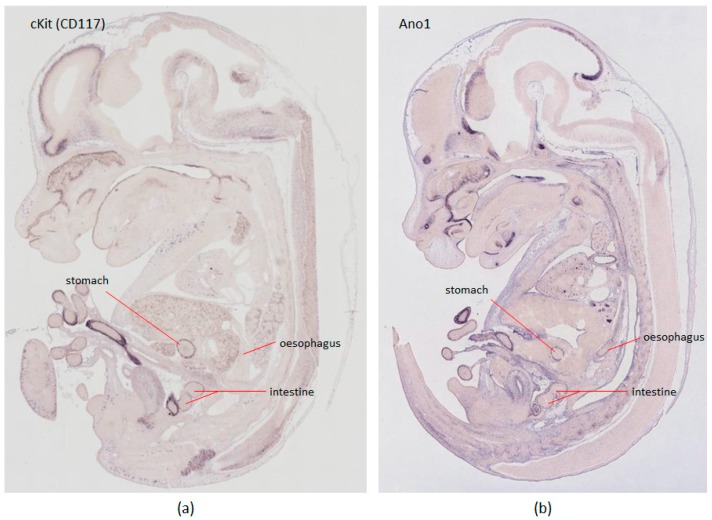
In situ hybridisation of *Kit* and *Ano1* genes in GenePaint [63]. The expression pattern of candidate genes in E14.5 mouse embryos was searched in the GenePaint database and compared. (**a**) cKit oncogene (GPID: MH1718) show moderate expression in gastrointestinal organs; (**b**) Gene Ano1 (GPID: MH710) shows strong expression within gastrointestinal organs, including the oesophagus. GPID: GenePaint Set ID.

The ICC marker of choice for the last 15 years has been Kit (CD117), a tyrosine kinase receptor, expressed on the plasma membrane of ICC cells [57]. Historically, the ACK2 Kit antibody clone has been popular for live cell analysis, however, the epitope this antibody detects is not stable under common tissue preparation methods, such as collagenase or paraformaldehyde treatment. Alternative Kit antibody clones, such as ACK-4 and D13A2, have proven to be more robust [58]. Moreover, Kit expression is not exclusive to ICC, and appears in several other cell types, such as melanocytes, hematopoietic cells, germ cells, and mast cells. As such, a more ICC specific marker, anoctamin-1 (Ano1), was recently proposed [59,60,61]. Ano1 is a calcium-activated chloride channel involved in the generation of the electrical slow wave, thereby displaying an important role in peristalsis of the GI tract [62]. Comparison of gene expression patterns of *Kit* and *Ano1* in E14.5 mice embryos from GenePaint database [63] are shown in Figure 2. Changes in the expression of Ano1 protein in ICC may contribute directly to abnormal slow waves and GI dysrhythmias [64]. Despite this specificity, a small group of Ano1^+^ Kit^−^ cells that was detected in the intestinal mucosa and submucosa did not resemble ICC. These Ano1-expressing cells were seen to reside at the base of mucosa crypts, but were morphologically similar to myofibroblasts [59]. Ano1 expressing cells have also been found in other tissues such as epithelia of foregut [65] and salivary glands [62]. Therefore, the double-labelling of cells using Kit and Ano1, when coupled with cell morphology, is thought to be a more reliable diagnostic approach for all ICC types in the GI tract of mice and humans than use of any single marker, and this approach is fast becoming the standard for ICC immunohistological identification [66]. Quantitative real time-polymerase chain reaction (RT-PCR) for transcripts of Kit (*Kit* gene transcript) and Ano1 (*TMEM16A* gene transcript) can also be used to check the purity of ICC isolation.

ICC are known to express a variety of receptors that help mediate motor neurotransmission; peptide receptors such as neurokinin (NK_3_) [34,67,68,69], somatostatin [70] and VIP receptors [31], M_2_ and M_3_ muscarinic receptors [31], and nucleotide receptors [71]. Expression of intracellular signalling intermediates, such as protein kinases [72,73] and cGMP [74,75], are expected in ICC-IM (or ICC-DMP), given their role as a neurotransmitter, but can also be found in pacemaker ICC-MY that do not appear to be direct targets of neuroeffector signalling [31,67]. Cultured ICC differ from freshly isolated ICC; cultured ICC express mesenchymal membrane markers [31] and have a high propensity to form networks of cells [76]. Only cultured ICC express smooth muscle myosin and VIP-2 [31]. Cultured ICC also express soluble and membrane-bound forms of stem cell factor (SCF, the maturation and development activation ligand for KIT), whereas freshly dispersed ICC only express soluble forms of SCF at the transcriptional level, suggesting the ICC in primary gut tissue requires cell-to-cell contact with other cells producing membrane-bound SCF (e.g., smooth muscle cells) in order to develop and maintain ICC phenotype (while cultured ICC are able to maintain ICC-MY network phenotype without other cells present) [31].

While Kit and Ano1 are accepted general markers of ICC, there are fewer markers for specific ICC subtypes. The ratio of Kit and Ano1 expression has offered a simple, but inconsistent, approach to identify ICC subtypes. For example, in the small intestine, Kit immunofluorescent staining is more intense in ICC-MY compared to ICC-DMP, whereas Ano1 staining remains comparable [58]. Neurokinin receptor 1 (NK_1_) transcripts are found in ICC-DMP/IM and a subpopulation of myenteric neurons, but not in ICC-MY; NK_1_ is thought to provide an opportunity to distinguish between some ICC subtypes [34,69]. The ligand for NK_1_, substance P, is a major excitatory enteric neurotransmitter in the GI tract. Exposure of gut muscle to exogenous substance P causes NK_1_ receptor to internalise in ICC-DMP/IM cells. This characteristic has been exploited in the separation of ICC-DMP/IM from ICC-MY, as further discussed immediately below. Overall, the ability to obtain large numbers of highly-purified human ICC from normal or diseased stem cell-derived gut organoids could provide a wealth of fundamental and clinically-relevant information regarding ICC phenotype and function in both health and disease.

## 5. Purification and Cell-Sorting Methods

Large-scale purification of ICC from primary tissue or bioengineered tissue has been problematic due to the difficulties in isolating these cells. ICC are distributed sparsely through the *Tunica muscularis* of the GI tract, and only make up a small proportion of all gut cell types. Various studies have attempted to harvest ICC from primary cultured gut samples and suspensions of freshly dispersed cells by aspirating visually identified native or immunostained Kit^+^ cells into micropipettes for RT-PCR or Western blot identification [31,77,78]. Whilst Kit labelling is not specific to ICC, the unique spindle-shaped and branching structures of Kit^+^ ICC make them clearly distinguishable by microscopy. Major drawbacks of these approaches are incorrect identification of non-specifically stained cells, experimental bias, inability to obtain sufficient mRNA for quantitative RT-PCR, or the inability to collect large number of ICC to be representative of all ICC population in GI tissue or to use them for large-scale cell analysis or transplantation. Table 2 compares the recovery of ICC from intestinal tissue using visual identification with high throughput techniques described in the following paragraphs. 

Fluorescence-activated cell sorting (FACS) has been implemented to isolate high numbers and purity of ICC from murine GI tissue [79]. The purification technique employs the labelling of *Tunica muscularis* strips with the ICC marker Kit (in this instance using the ACK2 antibody) conjugated with Alexa 488, followed by cell dissociation. A cocktail of antibodies conjugated with the Tri-colour fluorochrome (a tandem conjugate of R-phycoerythrin and Cy5) against other cells was also included, that stain with Kit antibodies, such as F4/80 and CD11b-expressing macrophage, CD11c-expressing dendritic cells, and CD45-expressing leukocyte cells. The labelled cell suspensions were sorted by FACS as clusters with green (presumed ICC), red and red + green fluorescence (single- or double-labelled macrophages and other hematopoietic cells), with unlabelled cells presumably including enriched populations of smooth muscle cells and myenteric neurons. This method of ICC-enrichment yielded 1.1–4.3% of presumed ICC from total cell population. Quantitative RT-PCR in the “ICC fraction” indicated a 62-fold increase of *Kit* expression relative to unsorted cells. However, CD68 expression also increased 5.6-fold in the “ICC fraction”, indicating that some cells that took up ACK2 were not detected by the macrophage label.

Magnetic affinity based pre-sorting with a CD45 column has also been implemented to increase selectivity, but at the expense of lower yields compared to FACS alone [79]. The same group also developed an immunomagnetic sorting (MACS) approach for ICC enrichment [80]. MACS allows for simultaneous sorting of large numbers of cells, advantageous when purifying rare cells, and improved recovery of intact cells with minimal stress during sorting compared to FACS. Primary cultures of murine GI muscles were labelled with Kit antibody (clone ACK-2) before cell dispersion and conjugation with magnetic beads for MACS separation. This technique yielded sufficiently pure ICC that retain electrical pacemaker activity after sorting, and the ability to be re-cultured despite a significant reduction in Kit expression. While MACS separation is less suitable for freshly dispersed cell suspensions, due to the presence of Kit^+^ macrophages, this is not an issue in organoid derived ICC, making MACS an attractive enrichment technique for stem cell-based ICC production.

An alternate approach that has been used to stratify ICC sub-types takes advantage of the cellular ability to internalise the NK_1_ receptor ligand, substance P [81]. NK_1_ is only expressed on ICC-DMP and ICC-IM, not ICC-MY, and when incubated with fluorescently labelled substance P, are bound and internalised by NK_1_. Chen et al. dissociated human jejunum circular muscle, and sorted by FACS, the resulting mixed cell population into a KIT^+^CD45^−^ ICC group, which was further stratified into ICC-DMP/IM (KIT^+^ substance P^+^) or ICC-MY (KIT^+^ substance P^−^) based on internalisation of fluorescent substance P. Incomplete labelling of all ICC-DMP/IM was noted, possibly due to poor penetration of labelled substance P, or non-uniform expression of NK_1_ receptor on ICC-DMP/IM cells, indicating that a more reliable approach would be beneficial. As there is currently no method for separation of ICC-DMP and ICC-IM cells in humans, more detailed information is needed regarding the gene and protein expression profiles of ICC. This is likely to be an iterative process, with stem cell-derived ICC potentially providing an attractive source of human cells with which to do this.

## 6. Functional Analysis

Analysis of ICC function is important for understanding the mechanisms of electrical rhythmicity in the GI tract, as well as ensuring organoid contractility are representative of mature gut motility. It is known that the conductances and “clock” mechanism possessed by ICC are important for generating slow wave peristalsis. Importantly, these functional properties have been shown to survive cell culturing [76], and they are observable in gut organoids [3,49,50,51]. Contractility is measured in organoids with a simple method of recording time-lapse changes in organoid diameter. Rhythmic slow wave activity indicates the presence of a functionally mature mesenchymal layer containing ENS and ICC networks [3]. Modulators of contractile activity (such as histamine to increase rate of contractility, and atropine to decrease contraction amplitude and frequency) have been applied to gut organoids, and shown to mimic motility responses observed in mature intestine.

Electrophysiology, such as whole-cell path-clamp recording of transmembrane action potential, has been used to detect spontaneous rhythmic currents as a measure of ICC physiological function within tissue or mixed cultures [78]. Cross sections of intestinal *Tunica muscularis* with the mucosa removed, can be pinned with glass microelectrodes and transmembrane potentials measured using a high input impedance amplifier [66]. Electrical recordings are made in the presence of nifedipine to reduce muscle contractions and maintain cellular impalements. Although ICC are connected to smooth muscle cells, and thus, the recorded activity could also be generated by smooth muscle cells, the branches of the ICC have been seen to withdraw upon electrode attachment, and the spontaneous activity was recorded from only ICC [78]. ICC-MY, expressing T-type Ca^2+^ channels, generate large amplitude, long lasting pacemaker potentials, while ICC-IM (isolated from the fundus which is devoid of ICC-MY) can also generate basic electrical rhythmicity (called unitary potentials), but do not regenerate and organise into slow waves [82].

Another method of detecting ICC functionality involves the use of rhod-2 imaging to detect oscillations in cytoplasmic [83] or mitochondrial Ca^2+^ concentrations [84,85], a critical step in the sequence of intracellular events that lead to electrical pacemaking.

Chemical inhibitors of specific cell types can be applied to confirm that the mechanical activity is regulated by ICC and the ENS. Introduction of the smooth muscle inhibitor l-type calcium channel blocker, nifedipine, should not affect contractions, as the pacemaker activity of ICC is insensitive to this [78], while methylene blue with light, known to injure ICC [86], should abolish all slow-wave components and worsen the regularity of the remaining electrical activity. The neuron blocker (TTX) would abolish spike action potentials. Some studies have argued that the contractility phenotype may be created during culture conditions, as ICC do not express smooth muscle myosin in situ [31]. Furthermore, when deprived of Kit signalling during in situ development, ICC cells take on a smooth muscle-like phenotype, including the expression of smooth muscle myosin [87]. Thus, it is possible that some contractile behaviour of organoid ICC is a consequence of culturing conditions. Whether or not this is the case needs to be explored further to confirm how closely organoid-derived ICC behaviour is similar to freshly dissociated and/or cultured primary ICC.

## 7. ICC Transplantation and Regeneration

The surgical transplantation of small bowel in mice has shown that newly integrated ICC networks are Kit^+^ within 3 days of transplantation, but a longer period is needed for the recovery of spontaneous contractile amplitudes to normal levels (about 30 days) [88]. The lag time for function to return was hypothesised to be a consequence of inflammatory changes in the intestinal muscle layers after transplantation. Gut contractility is known to be disrupted during intestinal infection by infiltrating immune cells that damage smooth muscle cells [41] along with a reduction in Kit^+^ ICC and increased ED2^+^ macrophages in the muscle layer [89]. ICC require the formation of gap junctions between each other, or with nerve fibres and smooth muscle cells [90]. It is likely that even after recovery of kit expression, the formation of gap junctions with other cell types is required for functionality. Sandgren et al. attempted murine transplantation of only autologous neurons and ICC to injured areas of the gut. Their results suggest successful transplantation of myenteric ganglia and ICC into host tissue, as evidenced by immunohistochemistry, though no follow up functional study was performed to determine improvements in contractility [91].

Development and maintenance of ICC network is dependent on Kit and SCF signalling [38,92]. The W locus is allelic for *Kit*, and a number of mutations of the W locus exist, in which the tyrosine kinase activity of kit is lost or compromised [93]. Mutations within the W locus, such as *W*/*W^v^* mutant mice, display reduced tyrosine kinase activity, and have a well-characterised loss or absence of ICC-MY in the small intestine, with a resultant loss of pacemaker activity [57,94]. Other mutants include *Sl*/*Sl^d^* mice (steel-Dickie mutant mice, in which the gene encoding the Kit ligand SCF is defective), and *Ws*/*Ws* rates (containing *Kit* gene mutation). Should a suitably large-scale and characterised source of ICC become available, these mutation mice could provide an excellent model system in which to test the validity of restoring ICC and pacemaker function in a region of the GI tract that lacks these cells and function.

A study published in 2013 demonstrated the potential of ICC replenishment therapy, using ICC networks generated following the co-culture of isolated ICC with explants of intestinal wall from *W*/*W^v^* mice [48]. Intracellular microelectrode recordings revealed the development of slow wave pacemaker activity following ICC transplantation in *W*/*W^v^* intestine cell cultures that were previously electrically quiescent and lacked any form of slow waves. The use of an allo-transplantation approach could provide a valuable means to restore Kit^+^ ICC into GI tissue in patients who are naturally devoid of these cells, or where ICC numbers have been reduced as a consequence of pathophysiological insult.

The transplantation of organoid-derived ICC has shown significant success with adult stem cell derived colon epithelium organoids and neurospheres regenerating damaged tissue. Colonic epithelium Lgr5^+^ organoids, containing all types of terminally differentiated intestinal epithelial cells, were dissociated and instilled into a colitis mouse model by enema. Within four weeks after transplantation, the donor-derived cells constituted a single-layer epithelium, which formed self-renewing crypts that were functionally and histologically normal [95]. Lindley et al. attempted regeneration of GI neurons with embryonic mouse neurospheres, and neonatal human neurospheres derived from bowel explant cultures and placed in the distal colon of embryonic aganglionic mice [96]. Coordinated contractions along the length of the implanted bowel were observed, despite the lack of fully differentiated ICC (noted by transmission electron microscopy). This study suggests that immature ICC can still generate pacemaker function. The contractility was observed with the presence of gap junctions noted between ICC and smooth muscle. Neuronal coupling is thought to occur in the developing gut due to close spatial relationship between neurons and ICC or smooth muscle cells. Although synapses were formed between transplanted neurosphere-derived neurons, no neuronal–muscular or neuronal–ICC synapses were seen. This may indicate that in neonatal mouse colon, neurogenic regulation of contractility is mediated by a neuromodulator, rather than by a classical synaptic neurotransmitter [97].

More recently, three studies have demonstrated the generation of enteric neural progenitors from human iPSCs, utilising them for transplantation in small animal models, or for in vitro modelling of Hirschsprung disease [2,98,99]. Fattahi [98] and Li [99] showed that enteric neural precursors could be generated from human iPSCs, and following transplantation into the colon of adult mice, the precursors migrated and generated enteric neurons. A similar concept of isolating ICC progenitors, for example, using the Kit^low^CD44^+^CD34^+^Insr^+^Igf1r^+^ phenotype used to obtain mouse ICC precursor cells [30] from organoids, and seeding them into damaged tissue, might allow for self-migration, alignment, and differentiation into specific ICC types in vivo, and accompanying restoration of gut motility.

## 8. Integration

A major question that has yet to be thoroughly investigated is the optimal route for delivery of the ICC to the affected regions of the gut. Clinical translation requires adequate and appropriate reinstatement of the ICC network. The method should be minimally invasive, but accurate and repeatable. With further research, it is possible that autologous (or allogenic) sources of organoid derived ICC cells could be identified and isolated in sufficient numbers to demonstrate the feasibility of this cell source for a cell-based therapy. Current transplantation focuses on localised injection of various cell types to the target area in the hope that environmental cues will drive them to differentiate into phenotypes appropriate for the location. Alternately, ICC networks and enteric neurons seeded and grown on a biosynthetic scaffold before transplantation might enable implant integration with the host tissue, through promoting an adequate blood supply for survival of the transplanted cells and promoting innervation for proper functionality.

When using cell-seeded scaffolds, the first step involves testing the ability of the scaffolds to support cell attachment, proliferation, differentiation and maintenance of cell phenotype and function. Collagen is the most commonly used natural material to tissue engineer the stomach and intestine. While collagen can promote cell adhesion and differentiation, it does not support cell alignment. Chitosan has been recently used as a natural material for human intestinal tissue engineering. Chitosan has been shown to maintain the phenotypes and the alignment of smooth muscle and enteric neural cells [100,101]; further testing is required to confirm ICC growth characteristics on chitosan.

Natural materials possess excellent biocompatibility, can be chemically modified and are found at low cost. However, they often exhibit poor mechanical properties that can be improved.

Some synthetic materials also represent strong candidates for cell attachment and transplantation due to their biocompatibility, biodegradability, mechanical properties, and ease of fabrication. At the 2015 American Gastroenterological Association conference, Rego described a method of incorporating ICC into bioengineered innervated smooth muscle embedded in hydrogel. Sheets of aligned smooth muscle cells were bioengineered in combination of ICC and neural progenitor cells. Contractility and response to electrical field stimulation was observed in the bioengineered sheets [102]. The limitation of synthetic polymers is the lack of effective binding domains, therefore, additional natural polymers have been investigated to enhance cell attachment and survival. Recent studies have utilised poly(l-lactide-co-caprolactone) (PLLC) scaffolds electrospun and immobilised with fibronectin, to enhance cell attachment and growth for oesophageal reconstruction [103]. Polyglycolic acid (PGA) that was coated with collagen and seeded with organoid units [104] regenerated epithelial and muscularis layers, and PGA coated with collagen and seeded with postnatally-derived progenitor cells was implanted in the omentum of mice for a period of 28 days [105]. In both cases, implantations in the mouse resulted in the generation of differentiated cell types of mature human GI tract.

Despite these initial successes, the degradation rate of cell scaffolds following implantation in the GI tract remains an issue. Physical and chemical modifications of the scaffolds help in ensuring a timely degradation following implantation, but the optimal material or set of materials is yet to be defined.

There has also been little emphasis on the trophic factor regimens that drive subtype differentiation and maintain phenotype. These factors need to be identified in vitro for incorporation in the next generation of cell-seeded scaffolds. Of particular interest are extracellular matrix-based micro-environmental cues, that have the capability of modulating both growth and morphogenetic signalling to the encapsulated ICC and gut cells. For successful tissue regeneration and development of GI function that mimics the native GI cells, neo-innervation must include neuron element network formation and connectivity. More fundamental research studying the interactions and communication of enteric neurons with peripheral neurons and ICC is required in order to be able to establish standards for neural connectivity. Stem cell-derived ICC offer a new opportunity to pursue these investigations.

## 9. Conclusions and Prospects

ICC are essential to coordinating smooth muscle function and generating slow wave contractility, necessary for normal gut function. Despite this, ICC regeneration as part of the bioengineering process has been largely ignored. Even through regenerative GI medicine is still in its infancy, major conceptual advances in this field have recently been achieved, particularly through the use of stem cell-derived gut tissue. The development of ICC organoid units in vitro, and the testing of cell-seeded scaffolds for intestinal regeneration in vivo, suggest that tools may now be becoming available, to link these two fields and open new avenues for investigating and potentially treating gut motility disorders. In the cases of motility disorders where ICC are specifically damaged, such a targeted cell therapy might be a simpler and more realistic solution compared to regenerating a whole gut segment. This review, outlining key studies that have established rudimentary frameworks for generating and purifying ICC from stem cell-derived gut organoids, provides useful insights into the potential for use of ICC in disease modelling and regenerative medicine over the coming decades.

## Figures and Tables

**Table 1:** Characteristics of interstitial cells of Cajal (ICC) subtypes.

Subtypes	Localisation	Morphology	Functions	Differential Markers	References
ICC progenitor	- Stomach - Intestine	Densely packed clusters of oval or circular cells extending within the *tunica muscularis*	Precursor ICC that can replenish damaged or lost ICC. Smooth muscle produced soluble stem cell factor (SCF) is responsible for its partial commitment into mature ICC	Kit^low^, CD44, CD34, InsR, IGF-IR	[30]
ICC-MY	- Stomach (antrum only) - Small intestine - Large intestine	Multipolar cells with branched processes connecting to each other and forming a network around the myenteric plexus in the space between circular and longitudinal muscle layers	- The dominant pacemakers in gastric muscle that generate slow-waves activity - ICC-smooth muscle coupling; electronically coupled via gap junctions or direct contact to propagate slow-waves from ICC to smooth muscle	Kit, Ano1, M_2_, M_3_, VIP-1, SCF-A, NK_3_	[9,12,31]
ICC-IM	- Distal oesophagus - Stomach - Pylorus - Small intestine - Large intestine	Bipolar or spindle-shaped cells associated with circular muscle (ICC-CM) or longitudinal (ICC-LM) muscle. Also occur in the connective tissue septa (ICC-SEP)	- Produce spontaneous depolarisations (unitary potentials) that generate low-frequency slow-waves - Mediate neural transmission between enteric nerves and smooth muscle - Stretch sensitivity in gastric muscles	Kit, Ano1, M_2_, M_3_, VIP-1, SCF-A, NK_1_, NK_3_	[15,31,32,33]
ICC-DMP	- Small intestine	Multipolar cells associated with the nerve bundles of the deep muscular plexus	- Mediate neural transmission in small intestine	Kit, Ano1, NK_1_, NK_3_	[15,34]
Others	- Pylorus (ICC-SM) - Large intestine (ICC-SMP)	Bipolar and multipolar ICC found in the submucosa (ICC-SM) and submucosal plexus (ICC-SMP) lie between the submucosal connective tissue and the innermost circular muscle layer	- Neurotransmission and pacemaker roles	Kit, Ano1,	[35,36]
Cultured ICC	- Cultures from freshly dissociated tissue	Primary culture consists of extensive network of multipolar ICC on the surface of smooth muscle cells. Secondary cultures contain a mix of multipolar and bipolar ICC. Networks of fibroblast-like cells and smooth muscle cells appear.	- Pacemaker properties generating contractility - A change towards a smooth muscle phenotype	Kit, Ano1, Smooth muscle myosin, M_2_, M_3_, VIP-1, VIP-2, SCF-A, SCF-B	[31]

IGF-IR, growth factor-I receptor; InsR, insulin receptor; M_2_ and M_3_, muscarinic receptor types 2 and 3; VIP-1 and -2, vasoactive intestinal peptides 1 and 2, NK_1_ and NK_2_, neurokinin receptor types 1 and 2, SCF-A and SCF-B, stem cell factors A and B.

**Table 2:** Summary of ICC recovered from gastrointestinal tissue.

Technique	Tissue/Total Cell Count	ICC Isolated	Reference
Visual identification	Mouse small intestine	38–38	[78]
Kit labelled FACS	Mouse small intestine 1.2–2.8 × 10^6^ cells	30,000–40,000	[79]
Immunomagnetic depletion of macrophages and Kit labelled FACS	Mouse small intestine 1.2–2.8 × 10^6^ cells	2000–4000	[79]
Kit labelled MACS	Mouse small intestine 0.86–1.12 × 10^6^ cells	7100	[80]
Kit and substance P labelled FACS	Mouse small intestine 15 × 1.5 mm strips	12,000 ICC-DMP/ICC-IM, 55,000 ICC-MY	[81]

## Abbreviations

ICC	Interstitial cells of Cajal
GI	Gastrointestinal tract
MY	Myenteric plexus
LM	Longitudinal muscles
DMP	Deep muscular plexus
iPSC	Induced pluripotent stem cells
ENS	Enteric nervous system
